# Newborn resuscitation: defining best practice for low-income settings

**DOI:** 10.1016/j.trstmh.2006.02.012

**Published:** 2006-10

**Authors:** Opiyo Newton, Mike English

**Affiliations:** a Kenya Medical Research Institute/Wellcome Trust Collaborative Programme, P.O. Box 43640, 00100 GPO, Nairobi, Kenya; b Department of Paediatrics, University of Oxford, Oxford, UK

**Keywords:** Resuscitation, Best practice, Meconium aspiration syndrome, Meconium-stained amniotic fluid, Low-income settings

## Abstract

Current resuscitation practices are often poor in low-income settings. The purpose of this review was to summarise recent evidence, relevant to developing countries, on best practice in the provision of newborn resuscitation. Potential studies for inclusion were identified using structured searches of MEDLINE via PubMed. Two reviewers independently evaluated retrieved studies for inclusion. The methodological quality of the selected articles was assessed using the Oxford Centre for Evidence-Based Medicine (CEBM) levels of evidence, whilst the Scottish Intercollegiate Guidelines Network (SIGN) grading system was used for subsequent recommendations. Based on available evidence, where there is meconium-stained liquor, routine perineal suction of all babies and endotracheal suction of active babies do not prevent meconium aspiration syndrome and have potential risks. Adequate ventilation is possible with a bag-valve-mask device and room air is just as efficient as oxygen for initial resuscitation. This review supports the view that effective resuscitation is possible with basic equipment and minimal skills. Thus, where resources are limited, it should be possible to improve neonatal outcomes through promotion of the effective use of a bag-valve-mask alone, without access to more sophisticated and expensive technologies. Basic, effective resuscitation should therefore be available at all health facilities and potentially in the community.

## 1 Introduction

Globally, over 5 million neonatal deaths occur each year. Birth asphyxia or failure to establish breathing at birth account for 19% of these deaths (Lawn et al., 2005a). In addition, birth asphyxia is closely associated with major neurodevelopmental sequelae such as cerebral palsy, cognitive impairment, epilepsy and chronic diseases later in life (Tan et al., 2004). Prematurity contributes another 28% to the total burden of newborn mortality (WHO, 2005), some due to inadequate respiratory support in the first minutes of life, whilst prematurity is also a risk factor for poorer outcomes in birth asphyxia (Costello and Manandhar, 1994). Perhaps less important, although still common, is the problem of meconium aspiration syndrome (MAS). Approximately 13% of liveborn infants are born through meconium-stained amniotic fluid (MSAF); of these, 5–12% develop MAS (Wiswell et al., 2000) manifesting as severe respiratory distress resulting in at best a requirement for careful, facility-based care and at worst death in 5–40% of cases (Fraser et al., 2005, Keenan, 2004, Wiswell et al., 1990). The implication of these statistics should be clear—that outcomes might be improved for more than 1 million infants per year through effective implementation of simple resuscitative techniques (Lawn et al., 2005a, Niermeyer et al., 2000).

However, whilst the fetal to neonatal transition during birth for all babies is marked by rapid and complex physiological changes critical to survival, most undergo a normal transition with only 5–10% requiring some degree of resuscitation at birth, ranging from simple stimulation to assisted ventilation (Tan et al., 2004). The problem for health workers and health systems is that it is often hard to predict reliably which pregnancies or labours will produce a baby requiring intervention. Reducing newborn mortality, whilst obviously depending on improved management of pregnancy and labour, therefore also requires that birth attendants should be able to provide basic newborn resuscitation (Bang et al., 2005). Such basic resuscitation, correctly done, is able to help most babies in need (WHO, 1997). However, even in health facilities, basic newborn resuscitation equipment may be missing, whilst health workers are often not adequately trained (English et al., 2004, WHO, 1997). The result is that observations of newborn resuscitation in developing countries often demonstrate poor practices (Kamenir, 1997). To a large extent, in many developing countries an inability to offer effective newborn resuscitation has been tolerated for many years. This largely reflects the belief that resuscitation is complex and dependent on the presence of (relatively) expensive technology making it ‘out of reach’ of low-income health systems.

However, emerging evidence suggests that resuscitation can and should be very simple without compromising the quality of the intervention (WHO, 1997). Following a simple A (airway), B (breathing) and C (circulation) approach it should be possible to provide resuscitation with simple equipment and minimal skills. The purpose of this review is to summarise the recent evidence, relevant to developing countries, that provides support for the view that effective newborn resuscitation can and should be provided in all health facilities and even perhaps in the community.

## 2 Methods

This review was aimed at answering the clinical questions on the ABC approach to newborn resuscitation included in Table 1. These questions emphasised topics that have major implications for the organisation and prioritisation of resuscitation services and the technology that supports resuscitation.

**Table 1:** Clinical questions

Steps	Clinical questions
A — Airway	(a) What is the value of routine perineal suction to prevent MAS in infants born through MSAF?
	(b) What is the value of routine endotracheal suction to prevent MAS in infants born through MSAF?

B — Breathing	(a) What is known about techniques of initiating ventilation during newborn resuscitation?
	(b) What is the preferred empirical resuscitation gas in asphyxiated term or preterm infants?

C — Circulation	(a) What is known about the use of chest compressions in newborn resuscitation?

D — Drugs	(a) What is the value of bicarbonate in correcting newborn acidosis during newborn resuscitation?
	(b) What is the value of adrenaline in newborn resuscitation?
	(c) What is the value of glucose in newborn resuscitation?

MAS: meconium aspiration syndrome; MSAF: meconium-stained amniotic fluid.

Potential studies for inclusion were identified by direct searches of the MEDLINE database via PubMed (1966–2005) by use of clinical queries (Haynes and Wilcynski, 2004, Montori et al., 2005). The following combinations of search terms were used:

• Meconium aspiration: (meconium aspiration OR meconium OR meconium aspiration syndrome) AND (suction OR suctioning) AND newborn resuscitation;
• Inflation breaths: resuscitation AND (inflation breaths OR breath^*^) AND (infant^*^ OR neonat^*^);
• Air vs. oxygen: (air OR oxygen) AND newborn resuscitation;
• Chest compressions: resuscitation AND (compressions OR chest compression OR compression rates) AND (infant^*^ OR neonat^*^);
• Sodium bicarbonate: acidosis AND (sodium bicarbonate OR bicarbonate) AND (newborn OR infant OR neonat^*^);
• Adrenaline: resuscitation AND (adrenaline OR epinephrine OR drugs) AND (newborn OR infant OR neonat^*^);
• Glucose: resuscitation AND (glucose OR dextrose OR drugs) AND (newborn OR infant^*^ OR neonat^*^).

The specific searches were aimed at identifying available evidence based on current randomised controlled trials (RCTs). However, where there were no RCTs, we briefly report findings based on previous literature reviews.

To ensure a comprehensive literature review, supplementary searches were conducted in the Cochrane Library, reference lists of all included articles and collections of experts in the field. The titles and abstracts of the retrieved articles were read by two independent reviewers and those that met the pre-set inclusion criteria were selected (see Appendix A).

The methodological quality of the selected articles was assessed using the Oxford Centre for Evidence-Based Medicine (CEBM) levels of evidence, which ranks the validity of evidence in a hierarchy of levels, with systematic reviews (SRs) as level 1 (strong evidence) and expert opinions as level 5 (weak evidence) (Phillips et al., 2001). Likewise, the grades of recommendations were based on the Scottish Intercollegiate Guidelines Network (SIGN) grading system, which places weight on the quality and body of evidence (Habour and Miller, 2001) (Appendix B).

### 2.1 Airway (A) (Table 2)

As soon as the umbilical cord is cut, if the baby is not already crying most authorities recommend that the baby is quickly dried with a (warm) towel for 10–15 s, re-wrapped in something clean and dry and placed where the baby can be kept warm. If the baby is now crying and active, an appropriate place is skin to skin on the mother's chest. If the baby is not crying after stimulation by effective drying or is not showing other signs of life, then resuscitation should begin. There is no place for more vigorous stimulation such as slapping the feet or bottom and as the baby is delivered no place for hanging the baby upside down to ‘drain out the liquor’.

**Table 2:** Airway

Reference/design	Country/setting	Inclusion criteria	Sample size	Intervention	LOE
(a) Meconium aspiration: perineal suction					
Vain et al. (2004); double-blind RCT	Multicentre, hospitals	Birth through MSAF of any consistency	2514	Suctioning of the oropharynx and nasopharynx before delivery of the shoulders (*n* = 1251) or no suctioning before delivery (*n* = 1251)	1b
		Gestational age ≥37 weeks			
		No abnormalities			

(b) Meconium aspiration: endotracheal suction					
Wiswell et al. (2000); RCT	Multicentre, hospitals	Birth through MSAF of any consistency	2094	Subjects were randomised to be intubated and suctioned (*n* = 1051) or to expectant management (*n* = 1043)	1b
		‘Vigour’ immediately after birth			

Linder et al. (1988); RCT	Israel, hospital	MSAF	572	Group I (*n* = 308): suctioning of the trachea under direct vision	1b
		Gestational age >37 weeks			
		Birth weight >2500 g		Group II (*n* = 264): no suctioning	
		Breathing spontaneously			

Daga et al. (1994); RCT	India, hospital	Babies born with thick meconium staining of amniotic fluid	49	Control group (*n* = 26) received only oropharyngeal suction, whilst the intervention group (*n* = 23), underwent oropharyngeal suction followed by tracheal suction	1b ‘–’

LOE: Oxford Centre for Evidence-Based Medicine level of evidence (May 2001); RCT: randomised controlled trial; MSAF: meconium-stained amniotic fluid; ‘–’: denotes a level of evidence that fails to provide a conclusive answer.

#### 2.1.1 Positioning

Failure to establish normal breathing at birth may result from the absence of a patent airway due to poor positioning of the head (or mechanical obstruction from blood, mucus or meconium). Thus, the first step for a baby should be to position the head in a neutral position to open the airway. The rationale and techniques for this are described in full elsewhere (Resuscitation Council (UK), 2000, Resuscitation Council (UK), 2001, WHO, 2006).

#### 2.1.2 Nasal and oropharyngeal suction on the perineum to prevent MAS

At the present time, delivery room management of babies born through MSAF includes suctioning of the mouth and nose before delivery of the shoulders. This addresses the concern that babies will inhale meconium present in the upper airway with their first breath, putting them at risk of MAS. The rationale for routine perineal suctioning of all babies born through MSAF has recently been questioned.

In a large study (*n* = 2514) conducted in Argentina and the USA, the effectiveness of intrapartum oropharyngeal and nasopharyngeal suctioning of term infants being born through MSAF was assessed. It was concluded that the procedure does not prevent MAS (Vain et al., 2004): there was no significant difference between treatment groups in the incidence of MAS (52 (4%) suction vs. 47 (4%) no suction; relative risk (RR) 0.9, 95% CI 0.6–1.3), the need for mechanical ventilation for MAS (24 (2%) vs. 18 (1%); RR 0.8, 95% CI 0.4–1.4), mortality (9 (1%) vs. 4 (0.3%); RR 0.4, 95% CI 0.1–1.5) or the duration of ventilation, oxygen treatment and hospital care. On the contrary, the procedure has been shown to be potentially harmful; it may cause apnoea and cardiac arrhythmias triggered by pharyngeal stimulation, worsening hypoxia and delay the establishment of adequate tissue oxygen delivery as well as risking damage to the upper airway (Linder et al., 1988, Vain et al., 2004).

#### 2.1.3 Endotracheal suction to prevent MAS

Tracheal suctioning of meconium in babies born through MSAF is an established intervention in many delivery rooms. However, recent evidence shows that this procedure might not be effective in reducing the incidence of MAS in active babies, i.e. those with good respiratory effort, heart rate >100 and reasonable tone.

Five studies have addressed the question of the efficacy of endotracheal suctioning of vigorous babies born through MSAF (Daga et al., 1994, Linder et al., 1988, Liu and Harrington, 1998, Wiswell et al., 2000, Yoder, 1994). Four studies (Daga et al., 1994, Linder et al., 1988, Liu and Harrington, 1998, Yoder, 1994), which compared vigorous term infants born through MSAF assigned to tracheal suction or routine care, showed no clear benefit of routine endotracheal suction. In subgroup analyses, this lack of benefit was apparent even in those born through very thick meconium (Yoder, 1994). On the contrary, the procedure was associated with an increase in minor pulmonary and laryngeal disorders (Linder et al., 1988). Similarly, in one large RCT (Wiswell et al., 2000), the incidence of MAS in the group assigned to tracheal suction was almost identical to the incidence in the group assigned to routine care without tracheal suction (3.2% vs. 2.7%, respectively). An additional Cochrane review that analysed four of the included studies (Daga et al., 1994, Linder et al., 1988, Liu and Harrington, 1998, Wiswell et al., 2000) came to similar conclusions (Halliday, 2000), although two of the studies (Daga et al., 1994, Liu and Harrington, 1998) had methodological flaws with randomisation and blinding of interventions.

### 2.2 Breathing (B) (Table 3)

#### 2.2.1 Assisted ventilation: initial inflation breaths

After airway opening, if the baby is not breathing, is breathing irregularly or shallowly, or is blue, modern recommendations are to give the baby five slow chest inflations with a bag-valve-mask (BVM) device (Resuscitation Council (UK), 2001). The basis for these inflation breaths is that until birth the lungs are filled with fluid. To clear the lung fluid, sustained application of pressures of approximately 30 cm of water for 2–3 s is required in a term infant (lower pressures are recommended in preterm infants). Too low a pressure may be ineffective in achieving expansion of lungs with initially low compliance, whilst too high a pressure may result in lung damage, notably pneumothorax. These slow inflation breaths are also intended to maximise opening of the more peripheral airspaces (Resuscitation Council (UK), 2000, Resuscitation Council (UK), 2001). Recent evidence suggests that such pressures can be reliably achieved with a BVM device in competent hands, at least in simulations (O’Donnell et al., 2005).

**Table 3:** Breathing (air vs. oxygen)

Reference/design	Country/setting	Inclusion criteria	Sample size	Intervention	LOE
Ramji et al. (2003); quasi-randomised	India, teaching hospitals	Asphyxiated babies	431	RAG (*n* = 210): asphyxiated neonates born on even dates given room air	1b ‘–’
		Weight >1000 g			
		Heart rate <100/min			
		Apnoea unresponsive to suction		100% oxygen group (*n* = 221): asphyxiated neonates born on odd dates given 100% oxygen	
		No abnormalities			

Vento et al. (2003); RCT	Spain, hospital, obstetric ward	Asphyxiated babies	151	RAG (*n* = 51) given room air	1b
		Term		Oxygen group (*n* = 55) given 100% oxygen	

Vento et al. (2001); RCT	Spain, hospital, outpatient clinic, obstetric ward	Asphyxiated	40	Room air (*n* = 19) or 100% oxygen (*n* = 21) via IPPV with bag and mask at 30 bpm	1b
		Term			
		Apnoeic, unresponsive to stimuli			
		Bradycardic			

Saugstad et al. (1998); quasi RCT	Multicentre, hospitals	Asphyxiated infants	609	Quasi-randomised by date of birth (even = room air (*n* = 288), odd = 100% oxygen (*n* = 331))	1b ‘–’
		Weight >999 g			
		Heart rate <80 bpm			
		Apnoea/gasping			
		No abnormalities			

Ramji et al. (1993); quasi-randomised	India, hospital	Birth weight >999 g	84	Neonates allocated to be resuscitated with either room air (*n* = 42) or 100% oxygen (*n* = 42)	1b ‘–’
		Apnoea			
		Heart rate <80 bpm			

LOE: Oxford Centre for Evidence-Based Medicine level of evidence (May 2001); RAG: room air group; RCT: randomised controlled trial; IPPV: intermittent positive pressure ventilation; ‘–’: denotes a level of evidence that fails to provide a conclusive answer.

Although supported by some experimental data and expert consensus, no RCTs were identified that have formally tested the value of inflation breaths when the baby fails to initiate breathing.

#### 2.2.2 Assisted ventilation: air vs. oxygen

Traditionally, 100% oxygen has been used to resuscitate all asphyxiated newborns irrespective of the severity of their condition. This practice largely reflects the concern that the poor tissue oxygen delivery that is a routine part of the fetal to neonatal transition is exacerbated by problems at or during birth, resulting in systemic and damaging oxygen deficiency (Vento et al., 2003). Thus, the aim has always been to correct any oxygen debt rapidly in the expectation that this will prevent or ameliorate any damage. Whilst apparently logical, emerging evidence suggests that this intuitive approach, which appears to restrict the delivery of effective resuscitation to sites with access to oxygen, is of no benefit. Thus, six studies (Ramji et al., 1993, Ramji et al., 2003, Saugstad et al., 1998, Saugstad et al., 2003, Vento et al., 2001, Vento et al., 2003), which compared resuscitation of asphyxiated newborns with room air or oxygen, found no differences between the groups with regard to mortality, morbidity and adverse neurodevelopmental outcomes. However, pooled analysis of an updated Cochrane review of four of the studies (Ramji et al., 1993, Ramji et al., 2003, Saugstad et al., 1998, Vento et al., 2003) showed a significant reduction in the rate of death in the group resuscitated with room air (RR 0.71, 95% CI 0.54–0.94; risk difference (RD) −0.05, 95% CI −0.08 to −0.01); number needed to treat 20, 95% CI 12–100) (Tan et al., 2004). These findings suggest, contrary to common current practice, that one death would be prevented for every 20 babies resuscitated with air rather than 100% oxygen.

Interestingly, room air resuscitation (RAR) has also been shown to have a number of short-term benefits not observed with resuscitation using oxygen. Recovery among RAR infants is quick as assessed by Apgar scores, time to first breath and onset of a sustained pattern of respiration (Ramji et al., 2003, Saugstad et al., 1998, Vento et al., 2001, Vento et al., 2003). Conversely, use of 100% oxygen is associated with hyperoxia and alterations in cerebral circulation (Vento et al., 2003).

However, some questions remain. In five studies (Ramji et al., 1993, Ramji et al., 2003, Saugstad et al., 2003, Vento et al., 2001, Vento et al., 2003), back-up oxygen was used for infants initially resuscitated with room air. However, this was allowed only for infants who remained cyanotic or bradycardic or in cases of resuscitation failure. Thus, there is a possibility that some subgroups of infants do require and benefit from resuscitation with oxygen. The effect on long-term development could also not be reliably determined because of methodological limitations in the one study that followed up infants beyond 12 months of age (Saugstad et al., 2003). Finally, the studies included few low birth weight/premature infants and thus the results cannot be extrapolated with confidence to this group. These topics should now be the subject of future specific trials that ideally improve on some of the methodological limitations encountered in the current data, including small numbers, inadequate long-term follow-up rates and lack of blinding of interventions/outcome measurements. Given these caveats, some caution should therefore be exercised in the application of the findings of the current studies to the whole population of babies requiring resuscitation.

### 2.3 Circulation (C)

As expected, there were no RCTs examining the value or delivery recommendations for chest compressions. Current guidelines are largely based on experimental animal data, simulation and expert consensus. The guidelines recommend compressions when bradycardia (heart rate <60/min and falling) persists despite adequate ventilation (Niermeyer et al., 2000, Resuscitation Council (UK), 2000). The compressions are required to deliver oxygenated blood primarily to the coronary circulation rather than the systemic circulation (in contrast to adult resuscitation, the heart is usually normal and just transiently ischaemic). Thus, compressions are only of value if one is certain that lung inflation has occurred (Resuscitation Council (UK), 2001). It is recommended that chest compressions should be co-ordinated with ventilations at a ratio of 3:1 to complete 30 cycles in 1 min, although the quality of chest expansion and compression is perhaps more important than attaining these rates.

### 2.4 Drugs (D) (Table 4)

Drugs have traditionally been used in newborns who do not respond to adequate ventilation and chest compressions (WHO, 1997). Clearly the emphasis is on adequate ventilation and compressions as drugs will be useless unless the primary problems of hypoxaemia/ischaemia are overcome and there is some degree of circulation (Resuscitation Council (UK), 2000, Resuscitation Council (UK), 2001). Although prominent in the minds of many, as well as evidence of their (inappropriate) prioritisation in practice in developing countries (Kamenir, 1997), there is little evidence for the value of drugs.

**Table 4:** Drugs

Reference/design	Country/setting	Inclusion criteria	Sample size	Intervention	LOE
Lokesh et al. (2004); Murki et al. (2004); RCTs	India, hospital, Intensive Care Unit	Asphyxiated infants continuing to need PPV at 5 min of life	55	Bicarbonate group (*n* = 27): given 4 ml/kg sodium bicarbonate over 3–5 min	1b
				Dextrose group (*n* = 28): given 4 ml/kg of undiluted 5% dextrose over 3–5 min	

LOE: Oxford Centre for Evidence-Based Medicine level of evidence (May 2001); RCT: randomised controlled trial; PPV: positive pressure ventilation.

#### 2.4.1 Sodium bicarbonate

Two small RCTs (Lokesh et al., 2004, Murki et al., 2004) that assessed the effects of sodium bicarbonate in asphyxiated neonates concluded that its administration does not help to improve survival or immediate neurological outcome. Similarly, a recent SR of base administration for preventing morbidity and mortality in preterm infants with metabolic acidosis found no statistically significant effect on mortality (RR 1.39, 95% CI 0.72–2.67; RD 0.12, 95% CI −0.12 to 0.36) or the incidence of intraventricular/periventricular haemorrhage (RR 1.24, 95% CI 0.47–3.28; RD 0.05, 95% CI −0.16 to 0.25) (Lawn et al., 2005b). The latter analysis reflected a concern that bicarbonate bolus may be associated with an increased incidence of intraventricular haemorrhage and hypernatraemia (Kamenir, 1997, Lawn et al., 2005b, Papile et al., 1978), although the volume/dose/concentration of sodium bicarbonate used may influence this outcome (Lokesh et al., 2004). The strength of the findings of this SR is, however, limited by the small number of studies (*n* = 2) and infants (*n* = 98) included. An additional review (Kecskes and Davis, 2002) found no evidence to support or refute the rapid correction of early metabolic acidaemia in low birth weight neonates.

These findings show that there is insufficient evidence to determine whether sodium bicarbonate infusion reduces morbidity and mortality or is even valuable in correcting acidosis in preterm infants with metabolic acidosis. Thus, further large randomised trials are needed.

#### 2.4.2 Adrenaline

Adrenaline (through α-adrenergic-mediated vasoconstriction) theoretically elevates the perfusion pressure during chest compression hence enhancing delivery of oxygen to the heart and brain. It may also enhance the contractile state of the heart, stimulate spontaneous contractions and increase the heart rate (Niermeyer et al., 2000). Evidence for a valuable role in practice in human newborns is, however, lacking. One SR on adrenaline administration to apparently stillborn or extremely bradycardic newborn infants found no studies on mortality and morbidity outcomes. Similarly, no studies on optimum dosage and route of administration of adrenaline were found. The authors concluded that the current recommendations for adrenaline use in newborn infants are based solely on evidence from animal studies and human adults. Thus, studies are required to determine the role of adrenaline in neonates, including those of extremely low birth weight (Ziino et al., 2002).

The consensus view is that adrenaline administration can be considered if bradycardia persists after a minimum of 30 s of chest compressions and adequate ventilation as judged by good chest movement (Niermeyer et al., 2000, Resuscitation Council (UK), 2001). However, this is clearly only possible if there are at least two skilled personnel present.

#### 2.4.3 Dextrose

There are no reported RCTs that have specifically examined the value of dextrose during newborn resuscitation. Therefore its role, if any, remains unestablished.

Expert consensus suggests that dextrose can be used if there is no response to adrenaline and sodium bicarbonate (Resuscitation Council (UK), 2001). The dextrose theoretically replenishes glycogen stores that are believed to diminish in the heart after delivery (Resuscitation Council (UK), 2001).

## 3 Implications for low-income settings

### 3.1 Environment/equipment

Newborn babies, especially small/asphyxiated babies, have difficulty in tolerating a cold environment. Continued exposure of the newborn to cold stress will result in a lower arterial oxygen tension and increased metabolic acidosis. To minimise evaporative heat loss, maintain a warm (25 °C) delivery room, keep all doors and windows closed, place the baby under a pre-heated radiant warmer for resuscitation if available and quickly dry the baby immediately after delivery (Niermeyer et al., 2000).

The minimum equipment needed for newborn resuscitation includes a dry towel, a bulb suction device and a bag and mask ventilator (Kamenir, 1997). A suggested list of resuscitation equipment is presented in Appendix C (Ministry of Health, 2002).

#### 3.1.1 Recommendations

Based on the findings of this review, the following recommendations can be made with regard to practices pertinent to the ABC approach to newborn resuscitation in low-income settings. The grades of recommendations were based on the SIGN grading system (Appendix B).  Environment

• To minimise evaporative heat loss, maintain a warm delivery room and dry the baby immediately after birth (Grade D).

  A — Airway

• Perineal suction is of no value and has potential risks. The practice should be stopped (Grade A).
• Routine endotracheal suction of vigorous term babies born through MSAF is of no benefit and may be harmful even if there is thick meconium (Grade A).
• Routine endotracheal suction in infants born through MSAF with poor tone/apnoea is of unknown value.
• Airway suction should be in response to evidence of obstruction and should be done under direct vision — deep, blind suction should be avoided (Grade D).
• If the baby is having difficulty in breathing, position the head in a neutral position to open the airway (Grade D).

  B — Breathing

• In cases of breathing difficulty, provide five inflation breaths each sustained for 2–3 s with a bag and mask ensuring that chest expansion is noted (Grade D).
• Infants can be initially resuscitated with room air just as efficiently as with oxygen (Grade A); spare oxygen should continue to be made available where possible in case of resuscitation failure.

Application of the above A (airway) and B (breathing) steps that require a minimum of equipment should be promoted at peripheral health facilities and ideally at the community level where a large proportion of births occur. Effective resuscitation (by one person) can be supplied in approximately 95% of newborns requiring intervention by following these two straightforward steps.  C – Circulation

• Chest compressions (after lung inflation) should be considered when bradycardia persists despite adequate ventilation (Grade D).
• Co-ordinate chest compressions and ventilations; they should be in a ratio of 3:1 with approximately 90 compressions and 30 breaths per min (Grade D).

To improve the outcomes of high-risk deliveries at health facilities, skilled performance of chest compressions should be supported through newborn resuscitation training.  D — Drugs

• The routine use of sodium bicarbonate to correct newborn metabolic acidosis does not help to improve survival or immediate neurological outcome. Bicarbonate infusion has potential risks. Thus, the practice should be stopped (Grade B).
• Administration of adrenaline is of no proven benefit but may be indicated if bradycardia persists after a minimum of 30 s of chest compressions and adequate ventilation as judged by good chest movement (Grade D).
• Dextrose is of no proven benefit but may be used if there is no response to adrenaline and sodium bicarbonate (Grade D).

It should be clear that there is little evidence for a useful role of drugs in newborn resuscitation. Therefore, their use should only be considered in delivery units with an adequate number of trained staff. Thus, only if adequate ventilation and chest compressions are being provided can drugs even be considered. Arguably, even if there are two resuscitators then the optimum approach would be for both to concentrate solely on the provision of effective approaches to A, B and C for at least 5 min, waiting for a third assistant before the use of drugs is considered.

#### 3.1.2 Research gaps

Based on the findings of this review, the following emerged as priority areas for future research in developing countries.

There is need for future trials:

• to establish whether community health workers or traditional birth attendants can be appropriately trained;
• to investigate the role, if any, of routine endotracheal suctioning in neonates born through MSAF who are not vigorous at birth;
• to determine the effects of room air or oxygen resuscitation on long-term neurodevelopmental outcomes in term and preterm infants;
• to confirm the postulation that some subgroups of infants could benefit from resuscitation with oxygen supplementation. The basis for this is post hoc findings of the use of back-up oxygen in cases of resuscitation failures in the RAR group; and
• to determine whether sodium bicarbonate infusion reduces morbidity and mortality in preterm infants with metabolic acidosis.

  **Conflicts of interest statement**

The authors have no conflicts of interest concerning the work reported in this paper.

## Figures and Tables

**Table:** 

	Patients	Interventions	Outcomes
Meconium aspiration	Preterm or term infants born through MSAF	(a) perineal suction vs. no suction	Mortality, morbidity, MAS, pneumothorax, HIE, intubation complications
		(b) endotracheal suction vs. no suction	
Air vs. oxygen	Asphyxiated preterm or term neonates	Comparison of air vs. oxygen	Mortality, morbidity, neurodevelopmental sequelae
Sodium bicarbonate	Preterm or term infants	Use of any dose/rate of sodium bicarbonate	Mortality, morbidity, neurodevelopmental disability

MSAF: meconium-stained amniotic fluid; MAS: meconium aspiration syndrome; HIE: hypoxic ischaemic encephalopathy.

## Appendix B Scottish Intercollegiate Guidelines Network (SIGN) grading system

  
**Grades of recommendations**

A At least one meta-analysis, systematic review (SR) or randomised controlled trial (RCT) rated as I++ and directly applicable to the target population; *or* a SR of RCTs or a body of evidence consisting principally of studies rated as I+ directly applicable to the target population and demonstrating overall consistency of results
B A body of evidence including studies rated as II++ directly applicable to the target population and demonstrating overall consistency of results; *or* extrapolated evidence from studies rated as I++ or I+
C A body of evidence including studies rated as II+ directly applicable to the target population and demonstrating overall consistency of results; *or* extrapolated evidence from studies rated as II++
D Evidence level III or IV; *or* extrapolated evidence from studies rated as II+

  
**Levels of evidence**

I++ High quality meta-analyses, systematic reviews (SRs) of randomised controlled trials (RCT), or RCTs with a very low risk of bias
I+ Well conducted meta-analyses, SRs of RCTs, or RCTs with a low risk of bias
I− Meta-analyses, SRs of RCTs, or RCTs with a high risk of bias
II++ High quality SRs of case–control or cohort studies. High quality case–control or cohort studies with a very low risk of confounding, bias, or chance and a high probability that the relationship is causal
II+ Well conducted case–control or cohort studies with a low risk of confounding, bias, or chance and a moderate probability that the relationship is causal
II− Case–control or cohort studies with a high risk of confounding, bias, or chance and a significant risk that the relationship is not causal
III Non-analytical studies, e.g. case reports, case series
IV Expert opinion

## Appendix C Recommended equipment for resuscitation of the newborn

  
**Essential**

• Source of heat, e.g. heater or lamp or Resuscitaire^®^
• Ambu bag (infant size)
• Warm dry linen
• Face masks sizes 0 and 1, preferably round
• A pair of scissors, strapping and tapes

  
**Priority**

• Source of oxygen, flow meter and tubings
• Suction equipment, i.e. rigid, large bore (Yankauer) suction tube and catheters, sizes F6, 8, 10
• Syringes and needles/swabs, preferably 1 ml, 2 ml, 10 ml
• Stethoscope

  
**Specialist use**

• Laryngoscope with extra batteries and bulb
• Laryngoscope blades sizes 0 and 1, preferably straight blade
• Endotracheal tube size 2.5 mm, 3.0 mm, 3.5 mm
• Umbilical catheters
• Nasogastric tube size F4 can be used as umbilical catheter
• Airway sizes: 000, 00, 0
• Adrenaline
• 10% dextrose
